# OntoGene in BioCreative II

**DOI:** 10.1186/gb-2008-9-s2-s13

**Published:** 2008-09-01

**Authors:** Fabio Rinaldi, Thomas Kappeler, Kaarel Kaljurand, Gerold Schneider, Manfred Klenner, Simon Clematide, Michael Hess, Jean-Marc von Allmen, Pierre Parisot, Martin Romacker, Therese Vachon

**Affiliations:** 1 Institute of Computational Linguistics, University of Zurich, Binzmühlestrasse, CH-8050 Zurich, Switzerland; 2 Novartis Pharma AG, NITAS, Text Mining Services, CH-4002, Basel, Switzerland

## Abstract

**Background:**

Research scientists and companies working in the domains of biomedicine and genomics are increasingly faced with the problem of efficiently locating, within the vast body of published scientific findings, the critical pieces of information that are needed to direct current and future research investment.

**Results:**

In this report we describe approaches taken within the scope of the second BioCreative competition in order to solve two aspects of this problem: detection of novel protein interactions reported in scientific articles, and detection of the experimental method that was used to confirm the interaction. Our approach to the former problem is based on a high-recall protein annotation step, followed by two strict disambiguation steps. The remaining proteins are then combined according to a number of lexico-syntactic filters, which deliver high-precision results while maintaining reasonable recall. The detection of the experimental methods is tackled by a pattern matching approach, which has delivered the best results in the official BioCreative evaluation.

**Conclusion:**

Although the results of BioCreative clearly show that no tool is sufficiently reliable for fully automated annotations, a few of the proposed approaches (including our own) already perform at a competitive level. This makes them interesting either as standalone tools for preliminary document inspection, or as modules within an environment aimed at supporting the process of curation of biomedical literature.

## Background

The growing body of published scientific findings within the domains of biomedicine and genomics poses, to research scientists and companies alike, the problem of efficiently locating the most relevant pieces of information. The research community is therefore keen to adopt novel text mining solutions, which have the potential of supporting such a discovery process [1]. Although there is a broad consensus regarding the need for text mining, there remains much ongoing debate on which of the many possible approaches are best suited for each specific goal.

Since its pioneering origins [2], the field of text mining in biomedical literature has seen steady growth, as indicated by a number of recent surveys [3-6]. In this research effort, an increasing role is being played by techniques originating in the field of computational linguistics [7-14].

In this report we describe experiments performed within the scope of the most recent BioCreative  competition (Critical Assessment of Information Extraction systems in Biology), using tools developed within the scope of the OntoGene project . BioCreative is ideally suited to create the conditions necessary for significant scientific advancement in the area of text mining, by providing a framework for testing and evaluation of research tools over shared tasks. In particular, three main tasks were defined by the BioCreative organizers: gene mention (GM), gene normalization (GN), and protein-protein interaction (PPI).

While GM focuses on detecting mentions of gene names in biomedical text [15], GN provides a more challenging scenario by requiring unambiguous gene identifiers rather than only mentions of gene names [16]. The PPI task was composed of four subtasks [17]: PPI-IAS (interaction article subtask; identification of abstracts that contains curatable protein-protein interactions), PPI-IPS (interaction pair subtask; identification of protein-protein interactions in abstracts), PPI-ISS (interaction sentence subtask; identification of sentences that provide evidence for protein-protein interactions), PPI-IMS (interaction method subtask; identification of the experimental method by means of which the interaction was verified).

The OntoGene project aims to develop and refine (semi-)automatic methods for the discovery of interactions between biological entities from the scientific literature. The OntoGene approach is based on dependency-based linguistic analysis of scientific articles [18]. As indicated by a number of recent publications [19-21], there is growing interest in dependency-based representations for the purpose of biomedical text mining. One of the advantages of a dependency-based syntactic representation is that it can be mapped easily into a semantic representation or, by application of simple transformations, can be used directly to match candidate answers with given queries, allowing easy identification of the arguments of complex relations [22].

The original goal of our participation was to test, in a standardized environment, the ability of our system to detect relations among biological entities, for example protein-protein interactions. Although our core target was the PPI-IPS task, we initially assumed that the interaction method would normally be mentioned close to the mention of the interaction itself, and therefore we decided to consider additionally the PPI-IMS task. Although our assumption soon proved to be invalid, in the meantime we did develop an alternative approach to the identification of experimental methods that proved to be very successful (best results in the official run of BioCreative).

The PPI-IPS can be decomposed into two major subproblems: identification of the protein in text and their normalization to UniProt identifiers (problem A), and selection of the valid interactions among the proteins identified in problem A (problem B). Problem A is functionally equivalent to various tasks of identification and normalization of biological entities (including the GN task of BioCreative). A number of tools have been developed [23-26], and some of them are freely available and can be used as components in more complex systems. Similar problems have been extensively tackled by the community in recent years. The identification of entries in text, before their normalization, can be seen as specific type of 'named entity recognition', as practiced since the MUC (Message Understanding Conference) competitions [27], or as a form of term identification. According to [28], the problem of term identification can be subdivided into three stages: term recognition (stage 1), term classification (stage 2), and term mapping (stage 3). In BioCreative stage 2 can be ignored because only one class of terms is of relevance (genes for the GN task, and proteins for the PPI tasks).

In the Biocreative GN task, participants have experimented various approaches to tackle this problem, which can basically be classified into two groups. The first type of approach is based on identifying all potential entity names in text (similar to the GM task), based on lexical, morphological and structural properties of the words, and later trying to match, disambiguate and filter those candidates against a shared resource such as UniProt [29,30]. This is equivalent to saying that stages 1 and 3 of term identification (as described above) are applied sequentially. The second type of approach is based on dictionaries or databases that include lists of potential entity names (and their corresponding identifiers) [31,32]. A dictionary look-up process, which automatically delivers also identifiers, is used to annotate candidates. This amounts to performing stages 1 and 3 of term identification in parallel. However, a disambiguation phase (based on local context) might still be necessary because many names will be ambiguous across multiple identifiers.

The identification of interactions (problem B, described above) is a less explored problem. One way to classify different approaches is to consider the amount of linguistic preprocessing that they apply to the input text. For example, systems like those described in [33-36] tend to ignore the syntactic structure of the sentences, which are treated just as word sequences. The identification of relations rests upon the frequent occurrence of given patterns (word sequences) between the two candidate proteins, possibly generalized by using part-of-speech tags, or some level of semantic tagging (for example, 'interaction verbs' are frequently used as indicators for a potential interaction) [37]. The patterns can be either automatically learned from annotated resources or manually constructed.

An alternative approach is to use a generic parser to build a syntactic structure for the sentences to be analyzed, which is then used to extract or confirm candidate interactions. Examples include use of the Link Grammar parser [11,38], an LFG (Lexical Functional Grammar) parser specifically adapted to Medline [14], use of an HPSG (Head-Driven Phrase Structure Grammar) parser [9,39], and use of the Stanford dependency parser [40]. Similar approaches were also used by [7,41,42].

Finally, there are some systems that are based on machine learning, using a number of linguistic features [43,44] and typically focusing on shallow syntactic parsing.

## Results and discussion

### Identification and selection of interactors

It is well known that protein names are highly ambiguous [45,46]. Researchers working in specific subcommunities tend to develop their own nomenclature, resulting in multiple names for the same protein. Acronyms and abbreviations further complicate the picture. Simply being able to recognize a protein name as such is just a starting point. The name needs then to be unambiguously qualified, by referring to an entry in a standard protein database, such as UniProt [47].

In order for that to happen, the disambiguation must occur at two levels: interspecies (to which specific organisms does the protein mention refer?) and intraspecies (within a given organism, which specific protein is meant?). For example, a protein mentioned in text as eIF4E could refer to a large number of different proteins. A search in the Swiss-Prot section of UniProt (the manually curated section) delivers 46 possible matches. However, if the term appears contextually with the mention of a specific organism, like in the sentence *'The Cap-binding protein eIF4E promotes folding of a functional domain of yeast translation initiation factor eIF4G1' *(PubMed: 10409688), then it is reasonable to assume that the name refers to a specific organism (yeast), thus restricting the possible matches in UniProt to the following two: EAP1_YEAST (eIF4E-associated protein 1) and IF4E_YEAST (eukaryotic translation initiation factor 4E). For the task of protein annotation, we have adopted a high-recall, low-precision term annotation approach, followed by very strict disambiguation steps, which gradually increase precision (at some expense for recall).

UniProt contains for each protein a list of frequently used synonyms, as well as the name and synonyms of its encoding gene. We have built a database that maps the synonyms to the protein identifier. We have further enriched such a list using morpho-syntactic rules that generate variants of the synonyms. Starting from the subset of UniProt delivered by the BioCreative organizers (which contained 228,670 protein identifiers), we extracted a list of 203,061 unique protein names (there are fewer names than IDs, because many names are ambiguous across species, especially due to orthologs), and - after generation of the variants - we obtained a database of 698,365 terms. Those terms are by necessity highly ambiguous; on average each term refers to three proteins, but there are also some terms that refer to hundreds of proteins.

The BioCreative organizers provided two text versions of each article, obtained by means of an automatic conversion from either the HTML or the PDF version of the article. Both of these conversions were found to be unsuitable for text processing, and therefore we decided early on to use only the abstracts, which we automatically downloaded, in plain text format, from PubMed (additional statistical information derived from the full articles was used only for the organism-based disambiguation). This strategy is based on the assumption that the authors will mention in the abstract the most relevant interactions that they discover (although this is by no means true in all cases). The input abstracts are tokenized using a custom tokenizer. The details of the tokenization are not important, but care must be taken that the same tokenization is used in processing the documents and the terminology list. The stream of tokens is then passed through a database look-up procedure, which tries to determine the longest possible match. As a result of the process, tokens forming terms are grouped together, and their multiple possible values as proteins are associated with them. As an example, the term eIF4E gets 46 different values, such as the following: IF4E_ASHGO, IF4E_RAT, IF4E1_SCHPO ... 4EBP2_HUMAN, 4ET_HUMAN.

Although in a few cases the results described in the articles apply to multiple species, in the majority of cases the article focuses on one (or in some cases two or three) organisms. In the training data, there were 449 articles with interactions involving only 1 organism, 142 articles with 2, 26 articles with 3, 6 articles with 4, 3 articles with 5, 1 article with 6, and 1 article with 9 different organisms (only 628 articles, among those distributed as training data, contained curatable interactions). Being able to determine with precision which is the organism used in the study leads therefore to a huge disambiguation effect.

The interactions normally involve two proteins from the same organism, but there can be cases of cross-species interactions that involve two proteins from different organisms. In the training dataset, out of 3,189 interactions, 396 (12%) are cross-species interactions. These cases are particularly problematic because they require local disambiguation of the protein.

The interactions between proteins belonging to different species can be classified in several categories.

The first category is linked to the medical field of infectious diseases, in particular disease-host interactions. Examples include interactions between viral and human proteins (such as between P53_HUMAN [human cellular tumor antigen p53] and VE6_HPV16 [protein E6 of the human papillomavirus variant 16; PubMed: 15175323]) and interactions between bacterial and human proteins (such as between CALM_HUMAN [human calmodulin] and CYAA_BACAN [calmodulin-sensitive adenylate cyclase of *Bacillus anthracis*; PubMed: 11807546]).

The second category is created by cloning and expressing one gene of species A in species B, for example interactions due to 'heterologous expression', such as between ISCS_ECOLI (cysteine desulfurase of *Escherichia coli*) and THTR_AZOVI (thiosulfate sulfur transferase of *Azotobacter vinelandii*; synonym: rhodanese-like protein; gene name: rhdA; PubMed: 16310786): *'The cysteine-desulfurase IscS promotes the production of the rhodanese RhdA in the persulfurated form. After heterologous expression in Escherichia coli, the Azotobacter vinelandii rhodanese RhdA is purified in a persulfurated form (RhdA-SSH). [...]'*

The third category contains interactions experimentally created to define the structure and the activity of some protein domains common to several species. For example, the *'motif-based search method to identify putative effector proteins' *(PubMed: 7493928), determination of a new protein family (PubMed: 9115257), comparison of the activity of homologous proteins (PubMed: 7890767), study of *'a pathway that is conserved from nematode to mammals' *(PubMed: 7657591), or an experiment designed to test the presence of *'homodimers' *(PubMed: 15131699).

For our experiments we have adopted a statistical approach based on the occurrences of mentions of organisms in the various sections of the paper. Just like for proteins, we have stored in our database a number of well-known synonyms for the organism (for example, *'murine' *is an adjective referring to MOUSE). Names and synonyms for organisms were automatically downloaded from NEWT . The relative frequency of species in the sections of the papers are combined linearly, with weights assigned through a learning procedure over a training corpus, and balanced by the known absolute frequency of species in biological research articles, which are derived from the training data. Interactions involving human proteins are more than 56.3% of the total, followed by mouse (9.3%), yeast (6.5%), *C. elegans *(6%), with each of the other organism represented in less than 5% of the interactions. Mentions in the abstract tend to have a predominant role in the balanced statistics.

The algorithm delivers a ranked list of species for each article. Such a list is then used to reduce drastically the number of possible interpretations for each term. The first step of disambiguation (organism-based) will simply go through all values for a term, and select those that match the best-ranked organism. If that fails to deliver any result, then it will proceed with the next organism, according to the ranking, until an assignment is found or a given threshold is reached.

Over the BioCreative training data (740 abstracts), the initial annotation step delivers 283,556 distinct protein values (precision = 0.0072, recall = 0.7469). (All P/R/F figures reported in this paper, unless explicitly noted, refer to the training data.) After the species-based disambiguation step, this number is reduced to 45,012 (precision = 0.0308, recall = 0.5763). The remaining ambiguity (intraspecies) must be resolved by other means.

With the collaboration of a domain expert, a small set of rules has been developed that reflects the typical naming conventions made by the authors. For example, the term MRGX, even if we know that it refers to a human protein, is still ambiguous among the following: MRGX1_HUMAN, MRGX2_HUMAN, MRGX3_HUMAN, and MRGX4_HUMAN. However, it is a typical convention that, if no further qualifiers are adopted, the term will refer to the first case (MRGX1_HUMAN). Alternatively, where there is a group of proteins characterized by Greek letter suffixes ('-alpha', '-beta', and so on), the convention is that the unqualified name usually refers to the '-alpha' variant (although exceptions are possible).

By sequentially applying the variant rules suggested by the domain expert, the second disambiguation step typically selects one value for each term. Over our collection of 740 abstracts, this reduces the number of protein candidates to 6,351 (precision = 0.1311, recall = 0.4974). As the figures reveal, one must accept a significant loss of recall at each disambiguation step in order to reach a minimally satisfactory precision.

The annotations of proteins and organisms obtained as described above were further augmented using the ALEx (Advanced Lexical Extractor) tool, developed in Research (NIBR) at Novartis, Basel, Switzerland. In ALEx the process of lexical extraction is mainly dictionary based. All terms that are recognized are semantically typed. Currently, ALEx covers concepts such as disease, company, product, gene, target, mode of action, and some more. The entire terminology for lexical extraction sums up to almost 1.7 million terms. The gene dictionary that we used consists of more that 150,000 normalized terms and nearly 0.5 million entries.

**Figure 1:**
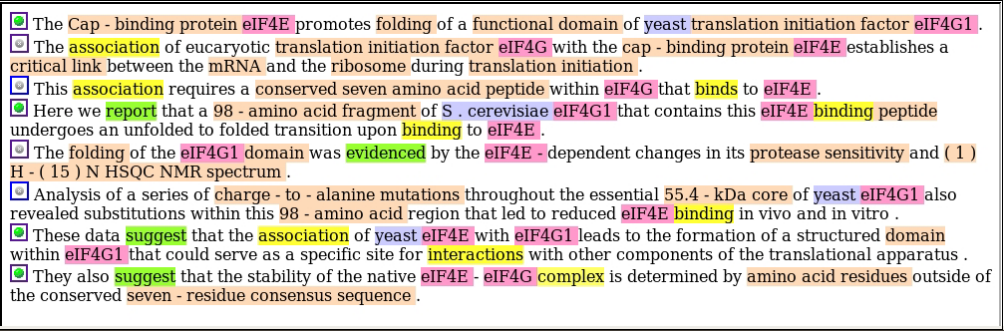
Example of an annotated abstract. The terms marked in violet are those identified by the system as protein names, the terms marked in blue are those identified as organism names, whereas those marked in orange are other classes of terms. Words marked in yellow are indicators for a relation, and words marked in green might suggest the presence of a curatable relation. The green dot on the left of a sentence indicates that the system considers that sentence as potentially containing a 'curatable' relation.

For the purposes of BioCreative, these additional entities do not need to be distinguished (they are all typed as 'OTHER'), but they are extremely helpful in the process of syntactic analysis of the sentences, as described in the next section. For an example of annotated abstract, see Figure 1.

**Figure 2:**
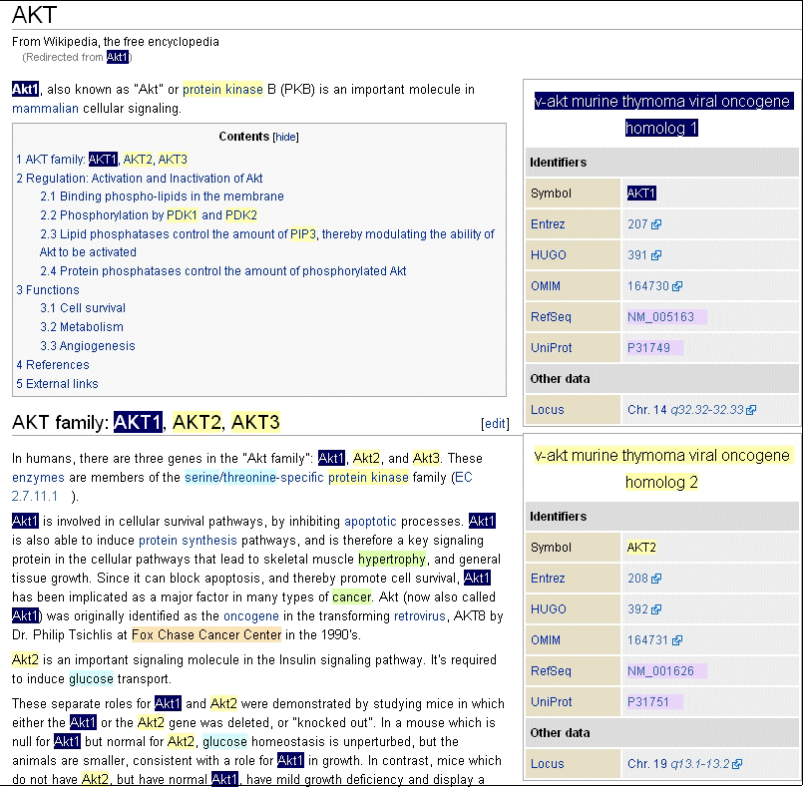
Annotation of a Wikipedia page by Ultralink. All genes are highlighted in yellow, diseases are depicted in green, products in light blue, companies in rose, and so on. Searching for all occurrences of AKT1 marks up all terms that refer to the corresponding normal form (for example, AKT1 but also *'v-akt murine thymoma viral oncogene homolog 1*').

At Novartis, the lexical extraction capabilities of ALEx are used for various application areas such as text mining, competitive intelligence, and data analysis for disease area, including the Ultralink system [48]. Figure 2 provides a screenshot of the entity recognition for Ultralink using the entry page for AKT in Wikipedia. Whenever a web page is loaded into a browser, its content is sent to the ALEx web service. The web service creates annotations by identifying terms and assigning a concept type to them. An additional display layer is added to the objects in the web page by highlighting the corresponding terms with a color code. Note that, internally, all terms in the texts are tagged with the normal form.

### Identification and selection of interactions

The BioCreative training set contains 740 articles obtained from either the IntACT [49] or MINT [50] databases, together with the 'gold standard' (the set of interactions that the curators have identified in each article as novel and relevant [3,189 interactions in total]). The average number of interactions per article is 4.31, but there are a few articles that contain unusually large number of interactions (the biggest number being 170). According to recommendations by the organizers, we dropped from the training set all articles containing more than 20 interactions. This left 719 articles, of which actually only 628 do contain interactions (for a total of 1,900 interactions, average 3.07 interactions per article).

Once reasonable values have been reached in the task of detecting proteins, the next problem to be tackled is that of identifying their possible interactions. A naïve approach would simply consist of generating all possible pairs of proteins mentioned in each single abstract. This results in a recall of almost 35%, but at the cost of an abysmal precision. Another simple approach consists in enforcing a maximal distance (in number of tokens) between any two mentions of the proteins. We have experimented with varying distances from 1 to 50 (without taking into account sentence boundaries), and found the best F measure value at the distance of 9 (precision = 0.0460, recall = 0.1765, F = 0.0729). The conceptually simpler (and more intuitive) approach of restricting the possible combinations to proteins within the same sentence, without requiring any maximal distance, delivers better results (precision = 0.0494, recall = 0.2077, F = 0.0798).

Still, although recall is relatively good (considering the limitations of the protein detection phase), precision appears to be too low for a practical application of the approach proposed. Therefore, a further filtering phase is required to select from among the proposed interactions only the relevant ones. In this respect, two kinds of false positives must be distinguished. On the one hand, there are pairs that correspond to interactions mentioned by the authors but that are not relevant to the curation task, either because they are well known interactions or because they play a secondary role with respect to the main interactions described. On the other hand, there are genuinely spurious protein pairs, which are not described by the authors as interacting but are simply a product of the simplistic way in which the pairs are generated. The strategies to filter out the false positives must therefore address both problems.

In the first case, the approach that we have followed is to try to identify in each abstract the sentences that describe the most relevant results according to the authors, and to distinguish them from the sentences that describe background results. An example of this could be the following: *'Previous studies have revealed a genetic interaction between DLG and another PDZ scaffolding protein, SCRIBBLE (SCRIB), during the establishment of cell polarity in developing epithelia.' *(PubMed: 11937021). An example of a sentence that reports 'curatable' results is the following: *'Here we report the isolation of a new DLG-interacting protein, GUK-holder, that interacts with the GUK domain of DLG and which is dynamically expressed during synaptic bouton budding.' *(PubMed: 11937021).

In order to distinguish between background and novel information, we adopted a machine learning approach based on a classifier, which takes as training data the lemmatized version of sentences whose status has been determined on the basis of the gold standard. A sentence is considered positive if it contains at least one pair of proteins belonging to one of the gold standard interactions for the abstract to which the sentence belongs (see Figure 1). After application of the 'novelty' filter, the results that we obtained on the training data are as follows: precision = 0.0945, recall = 0.1992, and F = 0.1282.

**Figure 3:**
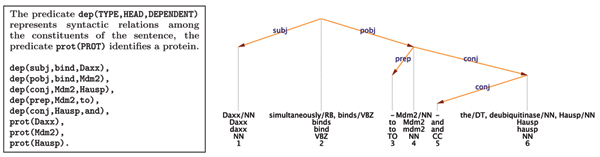
Dependency tree. Presented is an example of dependency tree (simplified internal representation on the left, graphical visualization on the right).

The second problem can be dealt with by taking into account the exact syntactic configuration in which the two proteins appear; does the context form a meaningful interaction? For example, in the sentence *'Daxx simultaneously binds to Mdm2 and the deubiquitinase Hausp.' *(PubMed: 16845383), three possible interactions can be considered (in the context of the BioCreAtIvE competition, the direction of the interaction is ignored): Daxx-Mdm2, Daxx-Hausp, and Mdm2-Hausp. However, on syntactic grounds (see Figure 3), only the first two interactions are licensed, whereas the third is not justified. We have developed a series of lexico-syntactic filters, which are applied in a cascade to each proposed interaction. The filters make use of lexical, morphological, and syntactic information delivered by a pipeline of NLP tools [51], including a novel dependency parser (for more details see [22]). For example, filters capturing the interactions shown in Figure 3 are (using a simplified notation):

int(X, Y):- dep(subj, H, X), dep(pobj, H, Y), prot(X), prot(Y).

int(X, Z):- dep(subj, H, X), dep(pobj, H, Y), dep(conj, Y, Z), prot(X), prot(Z).

**Figure 4:**
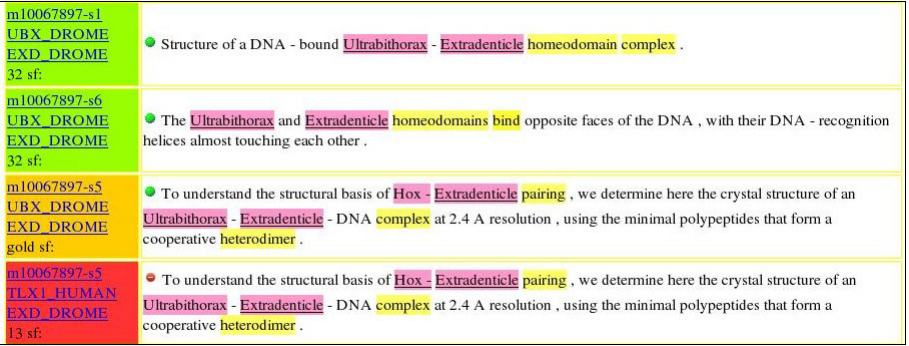
Support tools for the validation of filters. To the left of each sentence is shown the target interaction (either from gold standard or derived by the system). Green means that the interaction detected by the system matches an interaction in the gold standard. Gold marks an interaction in the gold standard not detected by the system. Red denotes an interaction detected by the system, but not contained in the gold standard. In other words, true positives are in green, false positives are in red, and false negatives are in gold.

Only if at least one of such filters applies to the specific case is the interaction considered further. The results that we obtain on the training data are as follows: precision = 0.5437, recall = 0.1839, and F = 0.2749. In order to enhance the usefulness and maintainability of the lexico-syntactic filters, a special type of visualization has been created (see Figure 4) showing, for each sentence and each interaction potentially contained therein, which of the filters captures the given interaction, including false positives and false negatives.

Our official results in the BioCreative challenge show that the approach is competitive, and can quite reliably extract interactions from PubMed abstracts. Although some of systems that process the full article do inevitably obtain better results (because not all the relevant interactions are necessarily mentioned in the abstract), our system achieved the fourth best results in the official BioCreative evaluation. Although the exact results vary slightly according to the type of evaluation performed (SwissProt only or full set of proteins, macro-average, or micro-average [17]), they consistently show that only systems that process the full text of the articles do better. For example, the evaluation based on all proteins, with results computed for each article and then averaged (macro-average), gives us the following results on the test corpus: precision = 0.2632, recall = 0.2484, and F = 0.2171.

The main reason why we focused only on abstracts is their ready availability in a standardized text format. It is conceptually simple (although time consuming) to create converters from the HTML version of the articles to a suitable text format. The main problem lies in the different structuring conventions of each journal, thus requiring a different converter for each format. Once the full articles have been converted in plain text, we could easily apply our tools, opening up the prospect of much higher recall.

### Identification of the interaction detection method

The original idea for this subtask was to compare two methodologies: pattern matching (supplemented by simple statistics) and machine learning. Because the resources for this subtask were extremely limited and time was running short, this comparison had to be postponed, and so only the results of the pattern matching approach were submitted. Pattern matching was conducted on a full-text version of the articles, because many abstracts do not mention all methods or any hints about them. These are normally mentioned in the 'Materials and methods' section. Therefore we converted the full HTML source version of the papers to text using the command "html2text -nobs". The quality of the result is not sufficient for syntactic parsing, as required by our approach to the PPI-IPS task, but sufficient for the pattern matching approach adopted for the PPI-IMS task.

The first important decision for this pattern matching approach was that, considering the limited resources and time budget, patterns for most methods could not be written by hand. So, we started with the method part of the PSI-MI (Proteomics Standard Initiative - Molecular Interactions) ontology [52] and took the official names, synonyms, and exact synonyms of the methods given there as baseline. These patterns were then supplemented by patterns automatically derived from the baseline patterns by considering several well known variations, such as insertion of spaces and hyphens (everywhere), deletion of spaces or hyphens (between words), interpolation of words (between words), truncation of heads, and so on. In this phase, just as in the next one, recall improvement was the primary goal.

The selection of methods for which patterns should be written by hand was based on the frequency of the methods in the training data and the recall and precision of the automatically derived patterns. Because just five methods account for two-thirds of all file-method pairs in the training data, these were carefully looked at by our team's biologist, who tried to find additional hints in some of the papers where the methods were not found using the automatically derived patterns. The method 'coimmunoprecipitation' (MI: 0019) and its hyponyms 'anti tag coimmunoprecipitation' (MI:0007) and 'anti bait coimmunoprecipitation' (MI:0006) were most successfully treated in that way, because they are extremely frequent in the training data and at the same time seldom recognized by the automatically derived patterns. After identifying files as containing one of the coimmunoprecipitation methods, the most important problem was the very low precision for most hints with good recall (for example, 'antibod' predicts 'anti bait coimmunoprecipitation' [MI: 0006] with recall 0.985 and precision 0.299) and the low recall for most hints with good precision (for example, 'flag-tagged' in combination with 'precipitat' predicts 'anti tag coimmunoprecipitation' [MI: 0007] with recall 0.434 and precision 0.543).

This could be overcome by a back-off algorithm, starting with the patterns with best precision (assigning their methods and excluding other coimmunoprecipitation methods), continuing with patterns with a lower precision (assigning their methods non-exclusively), and ending with a default (MI: 0019).

Similar approaches for 'pull down' (MI: 0096) led to much less improvement because the results for the automatically derived patterns were already rather good. This was even more so for the fifth method, 'two hybrid' (MI: 0018), and so the handcrafted patterns for this method were abandoned.

'Imaging techniques' (MI: 0428) was selected for a handcrafted pattern because recall was very bad. It was improved significantly by deriving the new pattern from obsolete method names, which must be mapped to MI: 0428 because they do not figure in PSI-MI 2.5 any more. An improvement in precision for 'biochemical' (MI: 0401) could be made by coupling the very imprecise pattern with other, more precise hints.

The pattern matching at this stage resulted in about 6.8 candidates per file with good recall (0.734) but bad precision (0.243). Obviously, the number of candidates had to be reduced to a degree comparable to the training data. For this, every candidate (method) was given a weight influenced by its frequency in the training data and the precision and recall of the patterns used to detect it.

The BioCreative evaluation framework allowed up to three runs to be submitted for each task. We decided on the following degrees of reduction: run 1, giving only the best candidate (and so the highest precision), was coupled with the results of the highest-precision run for subtask 3.2; run 2, giving the three best candidates (for best recall) was coupled with the results of the highest-recall run for subtask 3.2; and run 3, giving the best F measure by selecting up to three best candidates (additional condition was that candidates 2 and 3 reached a minimum in frequency and precision) was coupled again with the results of the highest-recall run for subtask 3.2. Because the interactants were identified in the abstracts only, whereas the methods were identified in the full text, no direct allocation of methods to specific interactant pairs could be achieved. Hence, we allocated every method for a file to all its interactant pairs.

Pattern matching just on the isolated 'Materials and methods' sections of the articles without candidate reduction yielded much higher precision than did the unreduced pattern matching of the full text, but after candidate reduction the results for the full-text pattern matching were slightly better.

The approach described has the advantage of being relatively efficient and self-contained, not requiring additional external tools for preprocessing the documents (save for the conversion into plain text). However, it might be effective only on articles that are thematically similar to those contained in the BioCreative training set. Moreover, experimental methods naturally evolve over time, as new methods are developed and older methods possibly disused, therefore limiting the application range of the tool. An extension to new methods, although relatively easy, requires the concomitant expertise of both a computational linguist and a biologist.

Nevertheless, in the official BioCreative evaluation, the tool achieved the best results (best precision = 0.6679, best recall = 0.5548, best F measure = 0.4836). Using the parent-node association evaluation measure, which includes the parent node of the expected method as correct answer (and therefore does away with some of the really complex disambiguation cases described above), the results jump to the following: precision = 0.6794, recall = 0.8548, and F = 0.6375.

As a service to the community, we intend to make this functionality available through a web server (integrated into the meta-server described in [53]). Given a PubMed identifier as input, the server will return a list of candidate methods identified in the article, ranked according to confidence.

## Conclusion

This paper presents an approach, directed at the extraction of protein-protein interactions from biomedical literature, based on sequential filtering of candidate interactions (pairs of proteins in sentences). The filters make use of linguistic information derived from a pipeline of NLP tools, in particular including a dependency parser. Furthermore, a pattern-based approach can recognize the most frequently used experimental methods with high reliability. The official results in the BioCreative competition show that the proposed approach is competitive.

## Abbreviations

ALEx, Advanced Lexical Extractor; GM, gene mention; GN, gene normalization; IAS, interaction article subtask; IMS, interaction method subtask; IPS, interaction pair subtask; ISS, interaction sentence subtask; PPI, protein-protein interaction.

## Competing interests

The authors declare that they have no competing interests.

## Authors' contributions

FR coordinated the BioCreative submission and wrote most of the manuscript. He also created tools for protein annotation and disambiguation and for syntax-based disambiguation. TK implemented the IMS approach, and wrote the corresponding section of the manuscript. KK created the XML-based pipeline and tools for visualization. GS is the author of the Pro3Gres parser. MK proposed the classifier-based approach used as a novelty filter for the sentences in the abstracts. J-MvA provided all biological insights. PP is the main creator of the ALEx system. SC is currently responsible for creating an XML-RPC-based annotation service for our IMS approach. MH, MR and TV provided various forms of support.
